# A Rare Case of Bartonella henselae Infective Endocarditis Causing an Embolic Cerebrovascular Accident

**DOI:** 10.7759/cureus.41364

**Published:** 2023-07-04

**Authors:** Kipson Charles, Andrew Abraham, Raghav Bassi, Rabab Elsadek, George Cockey

**Affiliations:** 1 Internal Medicine, University of Central Florida College of Medicine, Graduate Medical Education/Hospital Corporation of America (HCA) Florida, North Florida Hospital, Gainesville, USA

**Keywords:** infective endocarditis, septic embolic stroke, non-bacterial thrombotic endocarditis, blood culture-negative endocarditis, bartonella endocarditis

## Abstract

*Bartonella* is a facultative intracellular Gram-negative aerobic rod that is an important cause of culture-negative endocarditis that only accounts for 3% of all infective endocarditis (IE) cases. Throughout the literature, there have been very few documented cases of an embolic stroke caused by *Bartonella* *henselae *(*B. henselae*) IE. Following a comprehensive review of the literature, it appears that only a small number of articles have reported on the correlation between cerebrovascular accidents (CVAs) and *Bartonella* IE. Here, we present a case of a 42-year-old male with a cerebral embolic event as a complication of *B. henselae* IE.

## Introduction

*Bartonella henselae* (*B. henselae*) is a Gram-negative bacterium that is primarily associated with cat scratch disease. However, the medical literature has also linked it to infective endocarditis (IE), a serious infection of the heart's inner lining and valves. *B. henselae* IE is a rare [1] but potentially life-threatening condition that can cause a variety of complications, including cerebrovascular accidents (CVAs) or strokes [1]. In this article, we will present a case of *B. henselae* IE complicated by a CVA, describe the different types of endocarditis, and explore the link between *B. henselae* IE and CVAs, including the risk factors, symptoms, diagnosis, and treatment options for this condition.

## Case presentation

A 42-year-old male with a history of hypertension, dyslipidemia, and diabetes mellitus was transferred to our facility as a stroke alert with symptoms consistent with an acute onset of aphasia and weakness in the right upper and lower extremities. The patient did not report to work that day; therefore, co-workers who found him at home activated emergency medical services. He was last known to be in his usual state of health by family members about 24 hours prior to the hospital presentation. His family history was non-contributory to his current presentation.

Objectively, the patient was afebrile with a heart rate of 89 beats per minute, a respiratory rate of 16 breaths per minute, and a blood pressure of 188/79 mmHg. His physical examination was significant for a right-sided hemiparesis, right homonymous hemianopsia, and a grade III holosystolic murmur. His detailed laboratory findings are listed in Table 1.

**Table 1:** Detailed laboratory findings during hospitalization MCV: mean corpuscular volume, MCH: mean corpuscular hemoglobin, MCHC: mean corpuscular hemoglobin concentration, RDW: red cell distribution width, PT: prothrombin time, INR: international normalized ratio, aPTT: activated partial thromboplastin time, BUN: blood urea nitrogen, eGFR: estimated glomerular filtration rate, AST: aspartate transferase, ALT: alanine transaminase, LDL: low-density lipoprotein, HDL: high-density lipoprotein, TSH: thyroid-stimulating hormone, IgG: immunoglobulin G, IgM: immunoglobulin M, Ab: antibodies, NAA: nucleic acid amplification

Lab Value	Result	Reference Range
White Blood Cell	6.5 × 10^9^/L	4.0-10.5 × 10^9^/L
Red Blood Cell	4.68 X 10^12^/L	4.63-6.08 × 10^12^/L
Hemoglobin	11.8 g/dL	12.0-15.5 g/dL
Hematocrit	36%	40.1-51.0%
MCV	76.9 fl	79.0-92.2 fl
MCH	25.2 pg	25.7-32.2 pg
MCHC	32.8 g/dL	32.3-36.5 g/dL
RDW	14.3%	11.6-14.4%
Platelet	176 x10^9^	150-400 x 10^9^
Neutrophils %	59.4%	34.0-67.9%
Lymphocytes %	30.7%	21.8-53.1%
Monocytes %	8.7%	5.3-12.2%
Eosinophils %	0.5%	0.8-7.0%
Basophils %	0.2%	0.2-1.2%
Immature granulocytes %	0.5%	0.0-0.4%
PT	12.1 seconds	9.0-12.5 seconds
INR	1.1	1.0-2.0
aPTT	27 seconds	21-35 seconds
D-dimer	1.90 mg/L	< 0.5 mg/L
Sodium	134 mmol/L	136-145 mmol/L
Potassium	3.5 mmol/L	3.5-5.1 mmol/L
Chloride	102 mmol/L	98-107 mmol/L
Carbon Dioxide	26 mEQ/L	21-32 mEQ/L
Anion Gap	9.5 mEQ/L	3.0-15.0 mEQ/L
BUN	13 mg/dL	7-18 mg/dL
Creatinine	1.35 mg/dL	0.60-1.30 mg/dL
BUN/Creatinine	9.6	9.3-24.4
eGFR	58	> 60
Glucose	95 mg/dL	74-106 mg/dL
Total Bilirubin	0.5 mg/dL	0.2-1.0 mg/dL
AST	27 units/L	15-37 units/L
ALT	16 units/L	16-61 untis/L
Alkaline phosphatase	76 units/L	45-117 units/L
Total Protein	6.1 g/dL	6.4-8.2 g per dL
Albumin	2.8 g/dL	3.4-5.0 g/dL
Globulin	3.3 g/dL	2.8-4.4 g/dL
Albumin/Globulin Ratio	0.9	1.3-2.8
Triglycerides	138 mg/dL	0-149 mg/dL
Total Cholesterol	86 mg/dL	< 200 mg/dL
LDL Cholesterol	37 mg/dL	100-159 mg/dL
HDL Cholesterol	21 mg/dL	40-60 mg/dL
Vitamin B12	1043 pg/dL	193-986 pg/dL
Folate	13.2 ng/dL	3.1-17.5 ng/dL
TSH 3rd Generation	0.663 uIU/mL	0.358-3.740 uIU/mL
*Bartonella henselae* IgG	> 1:2560 A	Neg: < 1:320
*Bartonella henselae* IgM	Negative titer	Neg: < 1:100
*Bartonella quintana* IgG	Negative titer	Neg: < 1:320
*Bartonella quintana* IgM	Negative titer	Neg: < 1:100
Q Fever Phase I Ab	Negative	Neg: < 1:16
Q Fever Phase II Ab	Negative	Neg: < 1:16
Syphilis Serology	< 0.2	0.0-0.9
Coronavirus 2019 NAA	Negative	Negative

**Figure 1:**
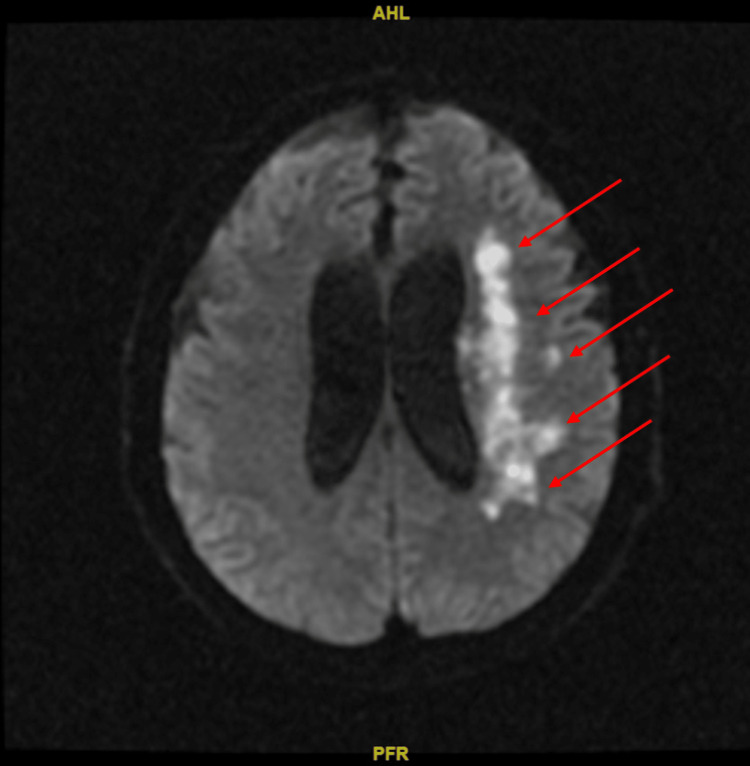
Brain MRI without contrast in DWI consistent with an acute, non-hemorrhagic infarct of the left MCA territory without midline shift (red arrows)

**Figure 2:**
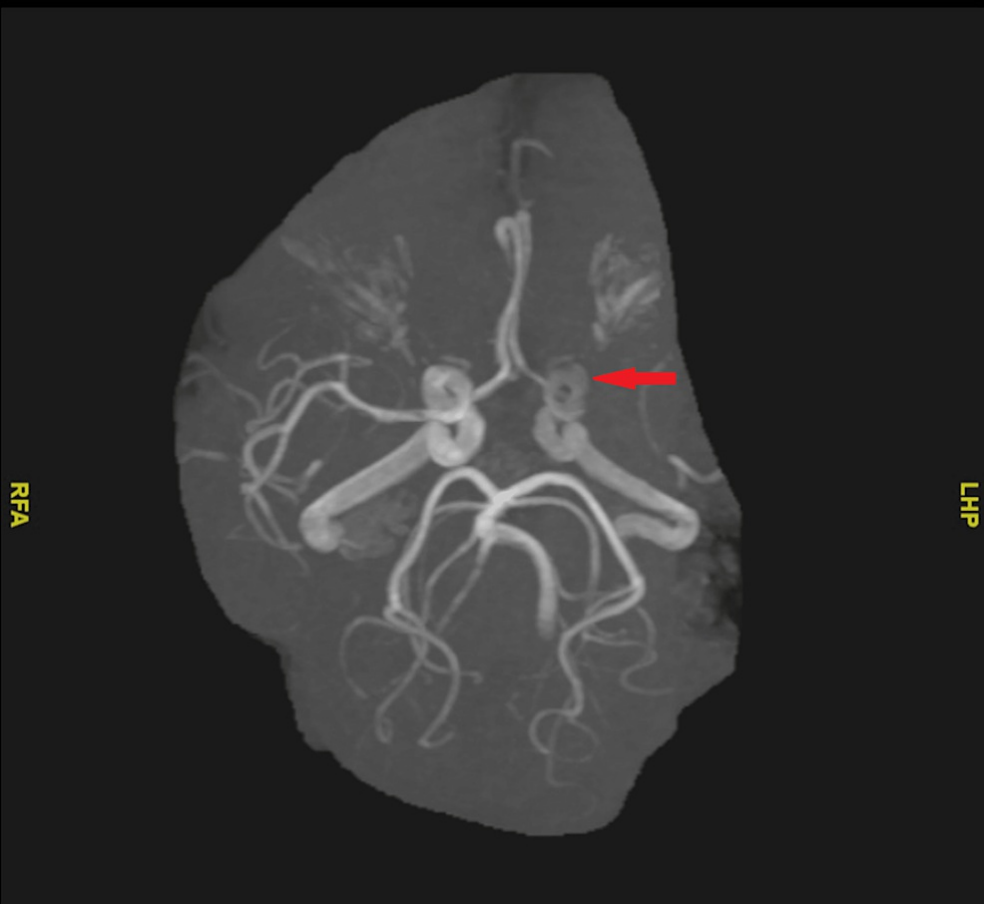
MRA of the head without contrast, demonstrating an absence of blood flow to the left MCA (red arrow)

**Figure 3:**
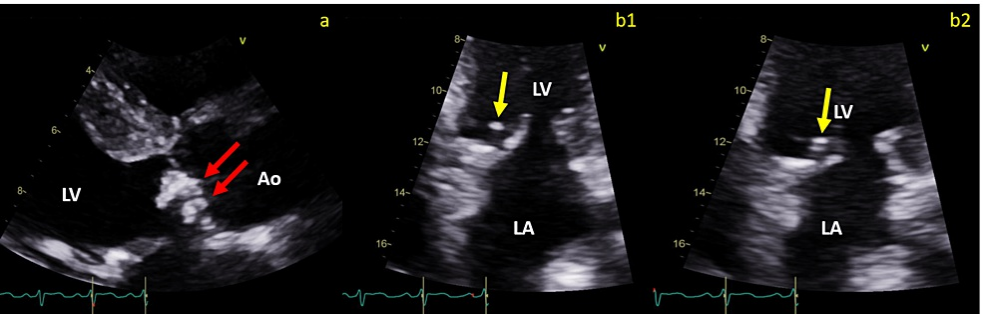
TTE with parasternal long axis view (panel a) showing severely thickened aortic valve leaflets with globular echodensities and mobile components involving the noncoronary and right coronary cusps, suggestive of vegetations (red arrows). The apical two-chamber view (b1-b2) demonstrated mildly calcified mitral valve leaflets with possible focal calcification, which could possibly be old, calcified vegetation (yellow arrows) LV: left ventricle, Ao: aorta, LA: left atrium

MRI of the brain showed an acute non-hemorrhagic infarct of the left middle cerebral artery (MCA) territory (Figure 1). Magnetic resonance angiography (MRA) of the head without contrast demonstrated an absence of blood flow to the left MCA (Figure 2). A transthoracic echocardiogram (TTE) revealed globular echodensities and mobile components attached to the aortic valve, which were suspicious of vegetations, with the largest one measuring 1.3 cm x 0.9 cm. The mitral valve also showed a small, calcified echodensity consistent with additional vegetations (Figure 3). The patient's relatives denied any record of mechanical heart valve implantation, intravenous drug abuse, or recent dental procedures. However, they mentioned that the patient had been living with cats for 13 years.

Intravenous ceftriaxone and vancomycin were administered as empirical antibiotic therapy after the collection of blood cultures, which eventually returned negative. Levels of B12, folate, TSH, LDL, coagulation panel, and viral panel were all unremarkable. The patient had a significantly elevated *B. henselae* IgG antibody titer of >1:2560. Vancomycin and ceftriaxone were then discontinued, and gentamicin 3 mg/kg/day along with doxycycline 100 mg daily were commenced for two weeks, followed by a 12-month course of oral doxycycline. The cardiothoracic surgery team communicated with the patient's family about the pros and cons of valve replacement in a case involving an acute embolic stroke. After considering the options, it was decided to conduct a follow-up with the patient in an outpatient setting after four weeks of hospital discharge. Following several days of physical and occupational therapy, the patient displayed gradual improvement and remained medically stable. The patient was discharged in an improved state and transferred to an outpatient rehabilitation center.

## Discussion

Endocarditis is a serious inflammation affecting the heart's inner lining and valves. It can lead to a variety of complications, including stroke, heart failure, and even death [1,2]. There are several types of endocarditis, each with its own characteristics and risk factors [1,3]. Types of endocarditis include infective bacterial endocarditis [1,2], which is further subdivided into acute bacterial endocarditis and subacute bacterial endocarditis; fungal endocarditis; non-IE; native valve endocarditis; and prosthetic valve endocarditis [1,3].

IE can be either a community or healthcare-acquired infection [4]. Community-associated IE occurs without recent exposure to a healthcare setting and is diagnosed within 48 hours of hospital admission. On the other hand, healthcare-associated IE (caused by *Staphylococcus* species and *Enterococcus faecalis*) develops within the context of recent contact with a healthcare setting and clinical manifestations appear more than or equal to 48 hours after hospitalization [5,6].

Epidemiology of IE

The overall incidence of IE ranges from 1.5 to 11 cases per 100,000 person-years [6-8]. The incidence of IE is difficult to accurately determine because case definitions have varied over time between authors and clinical centers [9]. Additionally, the incidence of risk factors such as rheumatic heart disease, degenerative valvulopathies, prosthetic valves, cardiac implantable electronic devices, or injection drug use varies over time, between regions and in low- and high-income countries [10].

While there are several causative organisms, staphylococcal species remain the most common isolated pathogen of IE in developed countries [7,10]. IE can also be caused by microorganisms such as *Candida *[10], *Histoplasma*, *Aspergillus*, and *Brucella*, although this is uncommon and typically occurs among individuals who abuse intravenous drugs [10,11].

The commonly forgotten culprits are the HACEK organisms, (*Haemophilus* species, *Aggregatibacter* species, *Cardiobacterium hominis*, *Eikenella corrodens*, and *Kingella* species), which account for about 3% of all cases of IE [8,12].

The diagnosis of IE can be challenging in some patients. Culture-negative endocarditis is even more difficult to uncover without a high index of clinical suspicion. Although it only accounts for 2-10% of all IE cases [12],* Bartonella*, the small facultative intracellular gram-negative aerobic rod, is a significant cause of culture-negative endocarditis [12]. Cerebral embolic events of cardiac origin, especially endocarditis, are associated with high morbidity and mortality as they frequently involve multiple sites of the brain [2]. When it involves culture-negative intracellular organisms such as *Bartonella* spp., the diagnosis of IE can often be delayed, which can lead to a dire prognosis [1].

Clinical presentation

The clinical picture of IE may be insidious, but fever, night sweats, new cardiac murmur, and weight loss are oftentimes the presenting symptoms [1,3,11]. Several hallmark signs may or may not be seen, such as Janeway lesions, Osler nodes, splinter hemorrhages, and Roth spots. The final diagnosis can be established using the modified Duke criteria (Table 2) [1,2]. Although the new onset of a murmur is arguably the most important clinical finding that represents significant morbidity, it can often be missed by clinicians, especially if right-sided IE is present [1].

**Table 2:** Major and minor criteria for diagnosis of IE according to Duke Criteria * viridans streptococci*,* *S. bovis*, HACEK group, or community-acquired *S. aureus* or *enterococci* Note: The diagnosis of definite IE requires either two major criteria, one major criterion and three minor criteria, or five minor criteria [1-3]

Major Criteria	Minor Criteria
Positive blood culture for typical IE microorganism or persistent bacteremia with typical IE organism*	Predisposing heart condition or IV drug use
Evidence of endocardial involvement (positive echocardiogram for IE or new valvular regurgitation)	Fever ≥38.0°C (100.4°F)
New valvular regurgitation	Vascular phenomena (arterial emboli, septic pulmonary infarcts, Janeway lesions)
	Immunologic phenomena (glomerulonephritis, Osler's nodes, Roth spots)

Culture-negative IE

Culture-negative IE can occur because of the previous administration of antimicrobial agents, inadequate microbiological techniques, and infection with highly fastidious bacteria or nonbacterial pathogens (e.g., fungi). The HACEK organisms were thought to be the most common agents of culture-negative endocarditis. However, recent studies have shown that HACEK organisms can be easily isolated using standard blood culture systems when cultured for at least five days [13,14]. Therefore, they are no longer considered an important cause of culture-negative IE. The most common causative agents of blood culture-negative IE are fastidious organisms such as zoonotic agents (e.g., *Bartonella* spp., *Coxiella burnetii*, or *Brucella* spp.), fungi, and *Streptococcus* species in patients who have received previous antibiotic treatment [13,14].

*Bartonella* spp.

Bartonellosis is caused by a wide range of *Bartonella* spp. Of the 30 different reported species of *Bartonella* [15], nine different species have been associated with IE [13,14], with *B. henselae*, *B. quintana*, and *B. bacilliformis* representing the highest prevalence [15,16]. Humans can become infected with *Bartonella* spp. through fleas, body lice, sand flies, or contact with flea-infested animals, most commonly cats and dogs (*B. henselae*) [15]. *B. henselae*, which is commonly known as "cat-scratch disease," can cause various clinical symptoms affecting the eyes, liver, brain, and heart. However, the initial indication of the disease usually manifests as a painless papule at the site of injury [15-17]. Painful lymphadenopathy in or around the affected area with possible suppuration, along with fever, fatigue, and weakness, can follow. Typically, these symptoms self-resolve; however, it is not uncommon to have a chronic infection with relapses [15]. Two of its most feared complications are peliosis hepatis and bacillary angiomatosis, which are commonly associated with HIV. The latter appears as one or several bright red-purple dermal nodule(s) that bleed on manipulation [15,17].

Detection of *Bartonella*

Given that many clinical laboratories have limited experience with culturing these bacteria, *Bartonella *spp. are challenging to culture in the laboratory, as they require specialized growth conditions, including the use of specific media and longer incubation periods, which can take several weeks to months to grow in culture [1,16]. As a result, the diagnosis of *Bartonella*-associated infections can be difficult and may require the use of molecular diagnostic techniques, such as polymerase chain reaction (PCR), serologic testing [1], and direct visualization with Warthin-Starry stain, a silver-based stain [15,16]. Clinicians should maintain a high index of suspicion for *Bartonella *spp. infections, particularly in patients with atypical clinical presentations and exposure to cats or other potential animal reservoirs [1].

Early recognition and appropriate treatment are important for improving outcomes for affected patients.

Complications of IE

IE involving the aortic valve is associated with a higher risk of embolic events compared to the involvement of other heart valves [1]. Aortic valve IE can cause complications such as septic emboli, abscess formation, and heart failure [1,16]. According to a review published by Brouqui and Raoult [1], the frequency of embolic events in IE can range from 10% to 50%, and there is a greater likelihood of such events occurring in patients with aortic valve involvement [2,14]. Individuals who have large vegetations, prosthetic valves, or staphylococcal infections are at a particularly elevated risk of embolization [18].

Transient ischemic attack and stroke compose a major cause of morbidity in IE, accounting for 40% to 50% of patients displaying clinical signs and symptoms of acute embolization causing neuro-vascular compromise [14,19]. Using clinical acumen, microbiology, physical exam, and imaging, such as transesophageal echocardiography and MRI, is required to diagnose septic embolic CVAs [2]. Furthermore, recently published data suggest that uncomplicated IE-related strokes (absence of meningitis, cerebral hemorrhage, or brain abscess) have a favorable prognosis when it comes to neurologic recovery [19].

Cerebral embolic events associated with *B. henselae* IE

According to medical literature, septic cerebral embolism affects approximately 40% of patients with IE [19]. Nevertheless, the occurrence of an embolic stroke caused by *B. henselae* is considered rare. Following a comprehensive review of the literature, it appears that only a small number of articles have reported on the correlation between cerebral vascular accidents and *Bartonella *IE [20-24]. However, that association should not be overlooked. It is important to consider *Bartonella *endocarditis in patients with stroke, especially those with a history of exposure to cats or other potential animal reservoirs, as well as those with a fever of unknown origin or culture-negative endocarditis, as it was initially assumed in this patient.

Management of *B. henselae* IE

Management of *B. henselae* IE involves a combination of antimicrobial therapy and surgical intervention in some cases. Because there are no randomized trials, there is insufficient data to make conclusive recommendations for treating *Bartonella *endocarditis. The existing literature is composed of case series and case reports [25-27].

For mild cases of bartonellosis, a single oral antimicrobial, preferably oral azithromycin 500 mg for 14 days, is sufficient for eradication [15,17]. According to a case report published in the European Journal of Case Reports in Internal Medicine [12] in 2019, patients with *Bartonella *endocarditis should receive a prolonged course of antibiotics to ensure complete eradication of the bacteria. The choice of antibiotic regimen depends on the severity of the infection, the presence of complications, and the susceptibility of the organism.

In general, a combination of IV antibiotics, such as gentamicin and ceftriaxone, for four to six weeks, followed by oral antibiotics, such as doxycycline and rifampicin, for several months, is recommended for *B. henselae *IE [12,15,26,28].

For *Bartonella *endocarditis, the first-line treatment is antibiotic therapy with oral or IV doxycycline 100 mg twice daily for up to three months, along with oral rifampin 300 mg twice daily as an adjunct for the first six weeks of antibiotic initiation. If unable to take rifampin, IV gentamicin 3 mg/kg/day for 14 days is an alternative agent; however, it is used cautiously due to the significant nephrotoxic side effect [15,28].

Despite antimicrobial therapy, surgical intervention, such as valve replacement, may be necessary for patients with severe valve damage, large vegetations, or refractory infection. An expert panel published in 2004 that was eventually incorporated into the 2005 American Heart Association Infective Endocarditis Guidelines [28] did not provide specific treatment recommendations for *Bartonella *endocarditis; however, it suggested that the duration of therapy should be tailored to the individual patient's response to treatment and that close follow-up with echocardiography is necessary to monitor for disease progression or recurrence.

## Conclusions

*Bartonella *IE is a significant cause of culture-negative IE and has been linked to ischemic stroke. Clinicians should be aware of this infection, particularly in patients with unexplained fevers, heart murmurs, and strokes. This is especially crucial when there is an epidemiological context suggestive of *Bartonella *involvement. The diagnosis of *Bartonella *IE can be challenging due to the lack of reliable tests, but serology and PCR may aid in the diagnosis. Future advancements in the field should focus on the development of more sensitive and specific diagnostic tools and optimal treatment strategies to improve clinical outcomes in patients with *Bartonella *IE and associated complications such as stroke. It is crucial to raise clinicians' awareness regarding the significance of comprehensive patient histories, which should include exploration of potential zoonotic vectors and reservoirs, in order to aid in the diagnosis of *Bartonella* IE and its associated complications, such as stroke. Furthermore, further research is warranted to advance our understanding of this infection and enhance patient outcomes.

## Appendices

Ab: Antibodies

ALT: Alanine transaminase

Ao: Aorta

AST: Aspartate transaminase

BUN: Blood urea nitrogen

dL: deciliter

DWI: Diffusion Weighted Imaging

eGFR: Estimated glomerular filtration rate

HDL: High-density lipoprotein

INR: International normalized ratio

IgG: Immunoglobulin G

IgM: Immunoglobulin M

L: Liter

LA: Left atrium

LDL: Low-density lipoprotein

LV: Left ventricle

MCA: Middle cerebral artery

MCV: Mean corpuscular volume

MCH: Mean corpuscular hemoglobin

MCHC: Mean corpuscular hemoglobin concentration

mEQ: milli-equivalent

mg: milligram

mmol: millimole

MRA: Magnetic resonance angiography

NAA: Nucleic acid amplification

ng: nanogram

pg: Picogram

PT: Prothrombin time

PTT: Partial thromboplastin time

RBC: Red blood cell count

RDW: Red cell distribution width

TSH: Thyroid-stimulating hormone

uIU: micro-international units
